# Poor Dietary Quality Is Associated with Low Adherence to Gestational Weight Gain Recommendations among Women in Sweden

**DOI:** 10.3390/nu12020317

**Published:** 2020-01-25

**Authors:** Hanna Augustin, Anna Winkvist, Linnea Bärebring

**Affiliations:** Department of Internal Medicine and Clinical Nutrition, Institute of Medicine, Sahlgrenska Academy, University of Gothenburg, 40530 Gothenburg, Sweden

**Keywords:** gestational weight gain, pregnancy complications, nutrition, dietary intake

## Abstract

Appropriate gestational weight gain (GWG) is important for fetal development and maternal health, but it is unclear what dietary factors predict GWG. The aim of this study was to investigate the association between dietary quality during pregnancy and GWG. In total, 1113 pregnant women were recruited when registering for antenatal care. GWG was defined according to the Institute of Medicine (IOM) guidelines. GWG was calculated as measured body weight at registration for antenatal care, to gestational week 37 ± 2. Dietary intake was assessed using a food frequency questionnaire (FFQ) administered in gestational week >31. In total, 40% gained within the IOM GWG recommendations, 25% had insufficient GWG and 35% excessive GWG. Women with a poor or fair quality diet gained approximately 2 kg more than women with a high-quality diet. Poor dietary quality was also associated with higher odds of excessive GWG, due to fat quality and intake of discretionary foods. In conclusion, poor quality dietary intake is associated with lower adherence to the guidelines on weight gain in pregnancy. A diet characterised by high-quality fat intake, low consumption of discretionary foods and high nutrient intake may promote healthy weight gain and prevent excessive GWG.

## 1. Introduction

Adequate gestational weight gain (GWG) is important for both fetal development and maternal health [1]. On the one hand, low GWG increases risk of infant small for gestational age (SGA) [2,3,4] and preterm delivery [1,5]. On the other hand, high GWG increases risk of infant large for gestational age (LGA) [2,3,4], preterm delivery [3], caesarean section (C-section) [5], maternal hypertension [1] and postpartum weight retention [2,6]. Both high and low GWG are associated with higher offspring fat mass during childhood [7]. In addition, high maternal GWG is associated with increased risk of offspring overweight and obesity [8].

GWG is regarded a greater contributor to adverse pregnancy outcomes than maternal body mass index (BMI) [3]. The risks associated with inadequate or excessive GWG are however modified by maternal pre-pregnancy BMI [9]. Therefore, the American Institute of Medicine (IOM) bases their recommendations on appropriate GWG on pre-pregnancy BMI. According to the IOM guidelines, recommended GWG is 12.5–18 kg for underweight women, 11.5–16 kg for normal weight women, 7–11.5 kg for overweight women and 5–9 kg for obese women [1]. Weight gain below or above the recommended range is considered inadequate or excessive.

Excessive GWG is increasingly common worldwide [1]. In Sweden, 36% of normal weight and 76% of overweight or obese women gain excessively during pregnancy [10]. This is of increasing concern since BMI in early pregnancy is increasing. From 1992 to 2016, mean early pregnancy BMI increased from 23.4 to 25 kg/m^2^ and the rate of overweight or obesity from 25% to 40% [11]. During the same time period, the rate of induced labour, caesarean section, and stillbirth increased, while the rate of infants with high birth weight (≥4500 g) and preterm delivery remained constant [11].

Dietary intervention during pregnancy may reduce the risk of excessive GWG by approximately 20% [12]. Data from Norway show that higher adherence to the New Nordic Diet, characterised by a high intake of fruit, vegetables, whole grains, potatoes, fish, game, milk and drinking water, is associated with optimal GWG and fetal growth [13]. While no association was seen between overall dietary quality and GWG in a US population [14], intake of specific foods (meat, dairy, fruit, vegetables, nuts) were associated with lower risk of excessive GWG [15]. Little is known about the effects of overall dietary quality on GWG. Additionally, very few data are available on nutritional intake among pregnant women in Sweden. Previous studies have found that reported dietary intake among pregnant Swedish women is of poor quality [16], with a low intake of many nutrients [17]. Neither of the previous studies have investigated associations between diet and GWG. We have previously shown, in a study of well-educated women, that higher intake of caloric beverages, snacks, fish and bread during pregnancy is associated with higher GWG [18]. To our knowledge, there are no population-based data published on dietary quality and GWG in Sweden. The aim of this study is therefore to investigate the associations between dietary intake quality and GWG in a population-based Swedish cohort. We hypothesise that poor dietary quality is associated with lower adherence to the IOM guidelines for GWG.

## 2. Materials and Methods

### 2.1. Recruitment and Inclusion

Pregnant women from the population-based GraviD cohort in the south-west of Sweden were included in this study. The cohort has been described in detail elsewhere [19]. In brief, 2125 women were included in the cohort when registering for antenatal care during autumn 2013 and spring 2014. In Sweden, antenatal care is free of charge to all pregnant women. All data collection was performed during routine visits throughout pregnancy. In the current analyses, inclusion criteria were singleton pregnancy and a provided food frequency questionnaire (FFQ). Women were excluded if they registered for antenatal care in gestational week >14, miscarried, delivered before gestational week 34, had missing data on pregnancy weight (*N* = 16), or implausible energy intake (<500 kcal/day, *N* = 7). A total of 1113 women were included in the analyses. All participants provided signed informed consent and all procedures were conducted in accordance with the Declaration of Helsinki.

### 2.2. Data Collection and Assessment of Dietary Intake

Maternal body weight was retrieved from medical records. Early pregnancy BMI was defined by the first measured body weight and classified as underweight (<18.5 kg/m^2^), normal weight (18.5–24.9 kg/m^2^), overweight (25–29.9 kg/m^2^), and obese (>30 kg/m^2^). GWG was calculated as body weight in gestational week 37 ± 2 weeks minus weight in gestational week ≤14. Those with complete weight data, or sufficient data to enable imputation, were included in the current analyses. If weight in gestational week 37 was missing, data were considered sufficient for imputation if there was a weight in gestational week ≤14, and in either gestational week 25 or 32. Imputation of total weight gain was performed using the mean GWG among women with the same early pregnancy BMI and the same GWG in week 25 or 32. This enabled the inclusion of an additional 58 women.

Data on obstetric outcomes were retrieved from medical records. The definitions of SGA and LGA were either weight or length at birth below or above 2 SD of the gender-specific population mean. Delivery by emergency C-section was defined as C-section after the onset of labor.

In the last trimester of pregnancy, all women were asked to answer an online semi-quantitative FFQ [20,21] that provided intake data on food levels, as well as macro- and micronutrient levels. The questionnaire has been validated among non-pregnant adults for macro- and micronutrients against 7-day food records and doubly-labelled water. In brief, reproducibility of nutritional intake was fair to high at *r* = 0.43–0.92 and validity was fair for intakes of most nutrients [20,21]. The FFQ took approximately 20 min to complete and specified dietary habits during the previous two months, thus reflecting intake in the second to early third trimester. A dietary quality index developed by the National Food Agency was used to determine dietary quality [22]. The index is designed to capture dietary quality (fibre, fat and discretionary foods) and compliance with dietary recommendations (intakes of fruit, vegetables, fish and whole grain bread). It scores intake of fruit and vegetables, whole grain bread, fish and shellfish, discretionary foods (sweets, cakes, soft drinks and French fries), margarine and butter, cheese and sausage, with a total min–max score of 0–12 points. Dietary quality was defined as poor (≤4 points), fair (5–8 points) or high (≥9 points). Positive contributors to the index are frequent intakes of fruit, vegetables, whole grain bread and fish. Negative contributors are frequent intakes of cheese, sausage and discretionary foods. Use of low fat margarine (≤40%) contributes positively while the use of high fat margarine or butter (≥60%) contributes negatively.

### 2.3. Statistical Analysis

Comparisons of nutritional intake depending on dietary quality (poor, fair and high) were performed using the Kruskal Wallis test. Associations between GWG and gestational outcomes SGA, LGA and emergency C-section were studied using logistic regression analysis. Associations between dietary quality and GWG were studied using linear and logistic regression analysis. All regression analyses were adjusted for gestational age at first visit (at first weighing), gestational age at delivery, maternal age, early pregnancy BMI, nulliparity, early pregnancy tobacco use, education level and pre-existing medical conditions (defined as renal, cardiovascular (including chronic hypertension), asthma or autoimmune medical conditions (diabetes mellitus, systemic lupus erythematosus or rheumatoid arthritis)). All analyses were conducted using SPSS Statistics version 25 (IBM Corp., Armonk, NY, USA). A total of 1113 women from the GraviD cohort were eligible for inclusion in this paper. Power calculations showed that this sample size would yield 77% power (alpha 0.05) to detect a 23% increased risk of excessive GWG among those with poor diet [23], (31% vs. 38% excessive GWG among those with high and poor quality diet, respectively). We assumed an overall 60% prevalence of poor dietary quality [16].

## 3. Results

### 3.1. Gestational Weight Gain

The characteristics of the women can be seen in Table 1. Mean (SD) GWG from registration for antenatal care to gestational week 37 ± 2 weeks was 13.6 (4.8) kg. In total, 40% gained within the IOM recommendations, 25% below and 35% gained more than recommended (Table 2). Mean GWG in kg for women with early pregnancy underweight, normal weight, overweight and obesity was 12.7 (4.9), 13.9 (4.4), 13.7 (5.3) and 11.7 (6.0), respectively.

Women with insufficient GWG had almost three-fold higher OR of having a child born SGA than women who gained within the recommendations (OR = 2.94, *p* = 0.004; Table 3). Similarly, women with excessive GWG had a nearly two-fold higher OR of LGA than those with adequate GWG, though this was not significant. Additionally, excessive GWG was associated with a nearly two-fold higher OR of delivery by emergency C-section, compared to adequate GWG (OR = 1.91, *p* = 0.02).

### 3.2. Dietary Quality and Nutritional Intake

Most of the women (54%) had fair dietary quality, 44% a poor quality diet, while only 3% had a high-quality diet. The nutritional intake of the women met the recommended daily intake for most nutrients (Table 4). However, only 10%–50% complied with the recommended daily intake of fiber, vitamin A, thiamine, vitamin E and potassium. The proportion who reached the average requirement of vitamin A, thiamine and vitamin E was 76%, 82% and 88%, respectively. Furthermore, ≤10% of the women complied with the recommended intake of saturated fatty acids, folate, vitamin D and selenium. The proportion who reached the average requirement of folate, vitamin D and selenium was 83%, 20% and 73%, respectively.

A higher dietary quality was related to higher intake of fibre (*p* < 0.001) and protein (*p* < 0.001), lower intake of total fat (*p* < 0.001) and saturated fat (*p* < 0.001) and higher intake of mono- (*p* = 0.020) and polyunsaturated fat (*p* < 0.001). Further, for most micronutrients, mean intake increased with increasing dietary quality (Table 4).

Linear regression analysis showed that, compared to women with a high-quality diet, those with poor (B = 2.286, *p* =0.013) or fair (B = 1.951, *p* = 0.031) dietary quality had a higher GWG corresponding to approximately 2 kg. Lower GWG was mainly associated with higher quality fat intake (B = −0.313, *p* = 0.007), but not with intake of fibre (B = 0.110, *p* = 0.418) or discretionary foods (B = −0.241, *p* = 0.267). Logistic regression analysis showed that poor and fair dietary quality were associated with higher odds of excessive GWG, compared to a high-quality diet (Table 5). This association seemed to depend mainly on higher-quality fat intake and a lower intake of discretionary foods.

## 4. Discussion

This prospective cohort study showed that 40% of the 1113 pregnant women gained within the IOM recommendations for GWG, 25% gained insufficiently and 35% excessively. Only 3% of the women had a high-quality diet. A poor or fair quality diet was associated with three to four times higher odds of excessive GWG, compared to a high-quality diet. Excessive GWG was associated with poorer fat quality and higher intake of discretionary foods.

Only 3% of the women in the current study were classified as having a high-quality diet. Median dietary quality score was 5, which is comparable to findings by Wennberg et al. who found a median score of 4 among pregnant Swedish women using the same index [16]. This previous smaller study assessed nutritional intake in the first trimester of pregnancy. It is unclear if the slightly higher median dietary quality in our study can be explained by chance, methodological differences or by better dietary habits later in pregnancy, when early pregnancy nausea and vomiting have subsided. The latter explanation is supported by previous research suggesting that these symptoms are associated with a higher intake of soft drinks and a deterioration in dietary quality [24,25]. The nutrient intake in the current study is similar to the intake in a recent study using the same FFQ among pregnant women in the north of Sweden [17].

The dietary quality assessed by the National Food Agency’s index seemed to be reflected in most nutrients, as nutritional intake was higher with increasing dietary quality. The recommended daily intake for most nutrients was met but only ≤10% of the women complied with the recommended intake of saturated fatty acids, folate, vitamin D and selenium. We have previously shown that almost 80% of women take nutritional supplements during pregnancy [26] and that many take the recommended folate in a multivitamin supplement. The contribution of supplements to overall micronutrient intake can therefore be substantial. Since the dietary intake of several micronutrients was low, the use of nutritional supplements during pregnancy may be warranted. However, overall dietary quality should also be improved as very few women were characterized as having a high-quality diet.

Women with a poor or fair quality diet had a significantly higher GWG than women with a high-quality diet, and they gained approximately 2 kg more. Further, poor dietary quality was associated with higher odds of excessive GWG, due to fat quality and intake of discretionary food. These findings are in line with results from Norway and the US, indicating that food choices are related to GWG [13,15]. Similar to previous findings [27,28], insufficient GWG in our study was most common among underweight women while excessive GWG was most common among women with overweight or obesity.

Limitations of this work include self-reported data on dietary intake, and that GWG was based on weights measured in early and late pregnancy, and weight changes before or after these time points cannot be ruled out. However, first trimester weight correlates well with self-reported pre-pregnancy weight, though pre-pregnancy weight is lower [29]. Consequently, early pregnancy weight is likely valid for ranking women according to their GWG. Still, an underestimation of the total GWG is probable. Furthermore, body weight was not measured in a standardised way and inconsistencies in clothing and time of day are likely. An additional limitation is the constant challenge in assessing dietary micronutrient intake with precision [20], and these results should be interpreted with some caution. The FFQ has only been validated in a non-pregnant population and its validity during pregnancy is uncertain. We have previously shown that the FFQ is valid for the estimation of dietary vitamin D intake among pregnant women [30], but the validation of other nutrients and dietary quality is warranted. The FFQ was administered in gestational week >31 and reflected dietary intake during the previous two months. Dietary intake in the first half of pregnancy, or after gestational week 31, is likely also important for GWG. Other limitations are missing information on factors that could impact GWG such as hyperemesis gravidarum, physical activity, dieting, thyroid hormone function and inter-pregnancy interval for parous women. Strengths include measured rather than self-reported weight and the relativity large cohort size. The GraviD cohort is population-based and while the FFQ was only available in Swedish, the FFQ respondents were similar to the overall cohort except for birth place; a slightly higher proportion was born in Sweden among the FFQ respondents (85%) than in the overall cohort (74%).

## 5. Conclusions

In conclusion, poor quality dietary intake is associated with lower adherence to the guidelines on weight gain in pregnancy. A diet characterised by high-quality fat intake, low consumption of discretionary foods and high nutrient intake may promote healthy weight gain and prevent excessive GWG.

## Figures and Tables

**Table 1:** Characteristics of the 1113 pregnant women from the GraviD cohort.

Continous Variables	Mean	SD
Gestational age at first visit (days)	57.9	13.0
Gestational age at delivery (days)	281.3	8.9
Gestational weight gain (kg)	13.6	4.9
Maternal age (years)	31.9	4.6
BMI at first visit (days)	24.1	4.0
Dietary quality index score (0–12 points)	4.8	1.9
**Categorical variables**	**%**	***N***
Parity		
0 children	42.5	473
1 child	42.4	472
2 children	12.2	136
≥3 children	2.9	32
Marital status		
Cohabitating with father	96.4	1068
Single	1.4	16
Other ^1^	2.2	24
Birth place		
North Europe	86.2	959
Continental Europe	5.1	57
Asia	5.7	63
Africa	1.4	16
America	1.6	18
Education		
Primary level	1.5	17
Secondary level	29.8	331
University level	68.7	764
Tobacco use in early pregnancy		
Yes	3.9	43
No	96.1	1070
Alcohol use in early pregnancy		
None	98.3	1043
≤1 time/week	1.7	18
Supplement use in third trimester		
Any	62.6	684
None	37.4	409

^1^ As stated in the medical records, and includes women who are living separate from the father, or who are in a same-sex relationship.

**Table 2:** Gestational weight gain according to the Institute of Medicines guidelines (1), among all women and per first trimester BMI category.

Gestational Weight Gain	All *N* (%)	Underweight (BMI < 18.5) *N* (%)	Normal Weight (BMI 18.5–25) *N* (%)	Overweight (BMI 25–30) *N* (%)	Obese (BMI > 30) *N* (%)
Insufficient	275 (24.7)	12 (46.2) ^a^	228 (30.9) ^a^	24 (9.4) ^b^	11 (11.7) ^b^
Adequate	449 (40.3)	11 (42.3) ^a,b^	344 (46.7) ^b^	71 (27.7) ^a^	23 (24.5) ^a^
Excessive	389 (35.0)	3 (11.5) ^a^	165 (22.4) ^a^	161 (62.9) ^b^	60 (63.8) ^b^
Total	1113 (100)	26 (100)	737 (100)	256 (100)	94 (100)

^a,b^ denotes non-significant proportions (*p* > 0.05) in gestational weight gain among the women grouped by first trimester body mass index (BMI).

**Table 3:** Associations between pregnancy outcomes and gestational weight gain, classified according to compliance with the gestational weight gain recommendations from the Institute of Medicine [1], among women in the GraviD cohort.

Gestational Weight Gain	SGA		LGA		ASEC	
	OR ^1^	*p*	OR ^1^	*p*	OR ^1^	*p*
Adequate (ref)	1.0		1.0		1.0	
Insufficient	2.94	0.004	0.17	0.090	1.33	0.386
Excessive	0.82	0.645	1.67	0.240	1.91	0.023

SGA, small for gestational age; LGA, large for gestational age; ASEC, emergency caesarean section. ^1^ Logistic regression model adjusted for gestational age at inclusion, gestational age at delivery, maternal age, early pregnancy BMI, parity, pre-existing maternal illness, tobacco use in early pregnancy and education level.

**Table 4:** Nutritional intake in pregnancy among the women in the GraviD cohort, assessed by a food frequency questionnaire.

Nutrient	RI ^1^	AR ^2^	All (*N* = 1113)	Poor Dietary Quality ^3^ (*N* = 486)	Fair Dietary Quality ^3^ (*N* = 597)	High Dietary Quality ^3^ (*N* = 30)	*p* ^5^
			Mean (SD)	Mean	Mean	Mean	
Energy (kJ)	-	-	7982 (3294)	7663 (3226)	8239 (3347)	8036 (2944)	0.001
Energy (kcal)	-	-	1908 (787)	1831 (771)	1969 (800)	1921 (704)	0.001
Carbohydrate E%	45–60	-	48 (6)	48 (6)	48 (5)	48 (7)	0.792
Fiber g/MJ	3	-	3 (1)	2 (1)	3 (1)	4 (1)	<0.001
Protein E%	10–20	-	16 (3)	16 (3)	17 (3)	18 (3)	<0.001
Total fat E%	25–40	-	33 (5)	34 (5)	32 (5)	31 (6)	<0.001
Saturated fat E%	<10	-	14 (3)	15 (3)	13 (3)	11 (2)	<0.001
MUFA E%	10–20	-	11 (2)	12 (2)	12 (2)	11 (2)	0.020
PUFA E%	5–10	-	5 (2)	5 (2)	5 (2)	6 (2)	<0.001
Vitamin A (RE)	800	500	752 (355)	655 (314)	823 (366)	903 (391)	<0.001
Thiamine (mg)	1.5	0.9	1.4 (0.6)	1.3 (0.6)	1.5 (0.7)	1.7 (0.6)	<0.001
Niacin (NE)	17	12	31 (11)	28 (10)	32 (12)	35 (11)	<0.001
Riboflavin (mg)	1.6	1.1	2.0 (0.8)	1.9 (0.8)	2.0 (0.8)	2.2 (0.8)	0.001
Vitamin B6 (mg)	1.4	1.0	2.0 (0.8)	1.7 (0.7)	2.1 (0.8)	2.4 (0.9)	<0.001
Folate (µg)	500	200	321 (133)	272 (115)	355 (131)	430 (156)	<0.001
Vitamin B12 (µg)	2.0	1.4	5 (2)	5 (2)	5 (2)	6 (3)	<0.001
Vitamin C (mg)	85	50	125 (68)	103 (61)	142 (68)	168 (56)	<0.001
Vitamin D (µg)	10	7.5	6 (3)	5 (2)	6 (3)	8 (4)	<0.001
Vitamin E (mg)	10	5	9 (4)	8 (4)	10 (4)	11 (4)	<0.001
Calcium (mg)	900	500	1064 (473)	1050 (490)	1079 (465)	976 (327)	0.351
Phosphorus (mg)	700	450	1420 (543)	1320 (522)	1496 (550)	1542 (472)	<0.001
Potassium (g)	3100	-	3143 (1110)	2814 (1036)	3386 (1096)	3610 (1157)	<0.001
Magnesium (mg)	280	-	349 (139)	306 (125)	381 (139)	417 (139)	<0.001
Iron (mg)	- ^4^	- ^4^	12 (5)	10 (5)	13 (5)	14 (5)	<0.001
Zinc (mg)	9	5	10 (4)	10 (4)	11 (4)	11 (3)	<0.001
Selenium (µg)	60	30	40 (16)	36 (14)	44 (16)	50 (15)	<0.001

RI, recommended daily intake; AR, average requirement; E%, energy percent; MUFA, monounsaturated fatty acids; PUFA, polyunsaturated fatty acids. ^1^ RI for pregnant women in the 2011–2012 Nordic Nutrition Recommendations. ^2^ AR for women in the 2011–2012 Nordic Nutrition Recommendations. ^3^ Dietary quality assessed by an index (score 0–12) designed to indicate intake of fibre, fat and discretionary food, where ≤4 points equals poor dietary quality, 5–8 points fair dietary quality and ≥9 points high dietary quality [22]. ^4^ Pregnancy requires 500 mg of stored iron. Supplementation is necessary for some women when dietary intake is insufficient to meet the extra needs. ^5^ Differences between groups were assessed by the Kruskal Wallis test.

**Table 5:** Associations between mid-pregnancy dietary quality and adherence to the Institute of Medicine guidelines for gestational weight gain (GWG) [1] among women in the GraviD cohort.

	Insufficient GWG		Adequate GWG		Excessive GWG	
	OR ^1^	*p*	OR ^1^	*p*	OR ^1^	*p*
**Dietary quality ^2^**						
High quality diet (ref)	1.0		1.0		1.0	
Fair quality diet	0.580	0.168	0.804	0.567	3.291	0.038
Poor quality diet	0.400	0.023	0.831	0.630	4.351	0.010
**Components of index ^3^**						
Fibre intake score (0–4 points)	1.043	0.536	0.961	0.499	1.012	0.849
Fat intake score (0–6 points)	1.225	<0.001	0.961	0.419	0.876	0.014
Discretionary food intake score (0–2 points)	1.050	0.645	1.126	0.202	0.818	0.053

^1^ Logistic regression models were adjusted for gestational age at registration in the first trimester, total gestational duration, maternal age, BMI in the first trimester, nulliparity, preexisting medical disorders, tobacco use in the first trimester and education level. ^2^ Dietary quality assessed by an index (score 0–12) designed to indicate intake of fibre, fat and discretionary food, where ≤4 points equals poor dietary quality, 5–8 points fair dietary quality and ≥9 points high dietary quality [22]. ^3^ Higher scores indicate higher dietary quality.
